# Home Blood Pressure Monitoring as an Alternative to Confirm Diagnoses of Hypertension in Adolescents with Elevated Office Blood Pressure from a Brazilian State Capital

**DOI:** 10.5935/abc.20170114

**Published:** 2017-09

**Authors:** Thaís Inacio Rolim Póvoa, Thiago Veiga Jardim, Carolina de Souza Carneiro, Vanessa Roriz Ferreira, Karla Lorena Mendonça, Polyana Resende Silva de Morais, Flávia Miquetichuc Nogueira Nascente, Weimar Kunz Sebba Barroso de Souza, Ana Luiza Lima Sousa, Paulo César Brandão Veiga Jardim

**Affiliations:** 1 Liga de Hipertensão Arterial - Universidade Federal de Goiás, Goiânia, GO - Brazil; 2 Escola Superior de Educação Física e Fisioterapia de Goiás (ESEFFEGO) - Universidade Estadual de Goiás (UEG), Goiânia, GO - Brazil

**Keywords:** Hypertension, Blood Pressure Monitoring, Ambulatory, Adolescent, Risk Factors

## Abstract

**Background:**

Regional differences of using home blood pressure monitoring (HBPM) as an alternative to ambulatory blood pressure monitoring (ABPM) in hypertensive adolescents are unknown.

**Objectives:**

Define if HBPM is an option to confirm diagnoses of hypertension in adolescents from a Brazilian capital with elevated office blood pressure (BP).

**Methods:**

Adolescents (12-18years) from public and private schools with BP > 90th percentile were studied to compare and evaluate the agreement among office BP measurements, HBPM and ambulatory BP monitoring. Office BP measurements, HBPM and ABPM were performed according to guidelines recommendations. Semi-automatic devices were used for BP measurements. Values of p < 0.05 were considered significant.

**Results:**

We included 133 predominantly males (63.2%) adolescents with a mean age of 15±1.6 years. HBPM systolic blood pressure and diastolic blood pressure mean values were similar to the daytime ABPM values (120.3 ± 12.6 mmHg x 121.5 ± 9.8 mmHg - p = 0.111 and 69.4 ± 7.7 mmHg x 70.2 ± 6.6 mmHg - p = 0.139) and lower than the office measurement values (127.3 ± 13.8 mmHg over 74.4 ± 9.5 mmHg - p < 0,001). The Bland-Altman plots showed good agreement between HBPM and ABPM.

**Conclusions:**

HBPM is an option to confirm diagnoses of hypertension in adolescents from a Brazilian state capital with elevated office BP and can be used as an alternative to ABPM.

## Introduction

Primary hypertension (HT) is no longer regarded as a rare phenomenon in childhood and adolescence.^1^ It is strongly related to obesity, a condition that continues to increase in young population, therefore HT prevalence will continue to grow among them.^2^ Blood pressure (BP) values are important markers in the evaluation of cardiovascular risk in adults,^3^ however, for children and teenagers there is scarce information regarding different BP measurement methods, and only in the last decade^1^ the interest in this subject has increased.

In Brazil, although many studies have assessed the prevalence of high blood pressure in adolescents in recent years, differences in measurement techniques and normalcy criteria according to regional differences make it difficult to know the actual prevalence. A systematic review of the literature found the prevalence ranging from 2.5 to 30.9%.^4^ The national representative ERICA study,^5^ evaluated 73.399 adolescents and identified a 9.6% prevalence of hypertension (values above the 95th percentile).

Investigate the viability and reliability of BP evaluation methods is necessary and contributes to clinical practice. For diagnosis, office BP measurements rank as the most common method and have a prognostic meaning for cardiovascular risk in adults. Nevertheless, BP values vary due to physiological and environmental stimulation, which indicates that a more accurate determination of BP values is needed. Identifying such variability may lead to more precise risk stratification, thus allowing early interventions initiatives.^6^

Taking multiple BP measurements within a short time period improves the reproducibility and increases the chances of obtaining accurate BP values. This repetition of measurements is possible with various BP monitoring methods, including ambulatory BP monitoring (ABPM), in which dozens of measurements are performed over a 24-hour period and is considered the gold standard,^7,8^ or home BP monitoring (HBPM), in which some measurements are performed over a few days throughout the week. The use of ABPM has limitations due to its higher costs, on the other hand HBPM which may be a potential diagnostic alternative, needs more investigation when used in adolescents, particularly considering regional differences.^6,7,9,10^ HBPM shows good viability if performed by the adolescents themselves or by a responsible adult with semi-automatic equipment and a specific protocol.^7^

The most common indication to use ABPM and HBPM in this particular subset of patients is for white coat hypertension (WCHT) diagnose, characterized by office BP measurement increased despite normal HBPM or ABPM values.^6,11^ Another indication is to detect masked hypertension, in which normal office BP is identified in patients with elevated HBPM or ABPM values.^9,11^

To increase scientific knowledge regarding BP measurement methods for adolescents considering regional differences, the objective of this study was to compare BP values obtained from office measurements, HBPM and ABPM and to evaluate the agreement among these methods.

## Methods

This was a cross-sectional study approved by the Research Ethics Committee of the institution (Register: 017/2010).

### Subjects

Adolescents aged between 12 and 18 years with altered BP (> 90th percentile for the respective age, gender and height)^1^ were identified by office measurement from a sample of 1025 young students from 26 schools. This was a representative sample of adolescents from a large city (1,302,001 inhabitants) in the Midwest of Brazil. Additionally, 33 normotensive adolescents were included. All subjects had an informed consent signed by their parents or legal guardians. The exclusion criteria were: physical handicap; pregnancy; chronic diseases (diabetes mellitus, kidney or heart disease); use of anti-hypertensive, antidepressants, anxiolytics, steroidal or non-steroidal anti-inflammatory drugs and contraceptives; and absence of sexual maturation (subjects with Tanner stages = 1).^12^

### Anthropometric evaluation

The anthropometric evaluation was performed using the standardization suggested by the World Health Organization.^13^ The measured variables were body weight, height and waist circumference. In addition, the body mass index (BMI) was calculated.

### Blood pressure measurements

#### Office measurement

Office measurements were performed by trained health professionals, based on the 4^th^ Task Force Technique.^1^ The procedure took place at the schools, in two different moments (one-week interval) and with two measurements (with a three-minute interval) at each time point. For the analysis, the mean of the second measurements was considered. We utilized OMRON, model HEM-705CP semi-automatic equipment, which was validated for use with adolescents,^14^ and cuffs in three different sizes (9x16 cm, 13x23 cm and 15x30 cm) were selected according to the adolescent’s right arm circumference (80 to 100%).

#### Home Monitoring (HBPM)

The same equipment, cuffs and techniques that were used for the office measurements were used for HBPM. Adolescents received the device at school and were told to perform two measurements (with three-minute intervals) during the day (between 06:00 and 10:00 a.m.) and two at night (between 06:00 and 10:00 p.m.) over 6 days, for a total of 24 readings. The overall mean value was considered for analysis.

### Ambulatory Monitoring (ABPM)

A *Spacelabs*® device model 90207 was used. The cuff size was the same of the office measurement and HBPM, and the exam was performed based on the American Heart Association technique.^15^ The equipment was programmed to perform one measurement every 15 minutes during the day (07:00 to 23:00) and one measurement every 20 minutes at night (11:00 p.m. to 07:00 a.m.). The adolescents were instructed to keep their arms relaxed during inflation/deflation and to return after 24 hours of monitoring with a report containing their primary activities during that period. Records in which at least 70% of the measurements were valid were accepted, and for the analysis, the mean of daytime obtained values was considered.

### Statistical analysis

Data were entered in duplicate and validated with Epi-Info (version 3.5.1), and the statistical analysis was performed with *SPSS* software (version 20.0; IBM Chicago, USA). The Kolmogorov-Smirnov test was used for data distribution evaluation and the paired Student’s t test for the comparison of systolic and diastolic pressure values between the methods. The continuous variables with normal distribution are presented as means and standard deviations. Pearson`s correlation coefficient was used to evaluate the correlation between the blood pressure measurements. Values of p < 0.05 were considered significant. We generated Bland-Altman plots^16^ to provide a visualization of the agreement between the measurements and a “mountain plot”^17^ to provide information about the distribution of differences between the methods. The ABPM method (daytime measurement) was subtracted from the other methods to obtain the mountain plots. The Bland-Altman and mountain plots were produced using *Medcalc software* (Version 12.7.0).

## Results

Among the 143 adolescents invited to participate the study, 133 (93%) accepted and 10 (7.0%) declined. No subject was excluded due to sexual maturation criteria. The final sample was composed of 133 adolescents, including 100 with altered BP and 33 normotensives. Overall, 63.2% were male with a mean age of 15 (± 1.6) years. characteristics.

HBPM presented mean SBP and DBP values that were similar to the daytime ABPM values and lower than the office measurement values. Office measurement presented higher mean values than those observed for daytime ABPM, and the correlation among the methods was moderate (Table 2).

**Table 2:** Comparison and correlation among office, home and ambulatory BP measurements (n = 133)

	Daytime ABPM	HBPM	p value*	r (p value)
SBP	121.5 ± 9.8	120.3 ± 12.6	0.111	0.70 (< 0.001)
DBP	70.2 ± 6.6	69.4 ± 7.7	0.139	0.60 (< 0.001)
	**Daytime ABPM**	**Office**		
SBP	121.5 ± 9.8	127.3 ± 13.8	< 0.001	0.60 (< 0.001)
DBP	70.2 ± 6.6	74.4 ± 9.5	< 0.001	0.45 (< 0.001)
	**HBPM**	**Office**		
SBP	120.3 ± 12.6	127.3 ± 13.8	< 0.001	0.75 (< 0.001)
DBP	69.4 ± 7.7	74.4 ± 9.5	< 0.001	0.53 (< 0.001)

Values expressed as the mean ± standard deviation. SBP: systolic blood pressure (mmHg); DBP: diastolic blood pressure (mmHg). r- Pearson's correlation test.

* paired Student's t test.

The overall mean of 24-hour ABPM BP was 118.3 ± 9.1 mmHg for SBP and 66.4 ± 6.0 mmHg for DBP, which were significantly different than the overall mean of HBPM (SBP, p = 0.009; DBP, p < 0.001) and the office measurement (p<0.001 for SBP and DBP). A strong correlation (r = 0.72, p < 0.001) was found between SBP from 24-hour ABPM and HBPM, whereas a slight correlation (r = 0.39, p = 0.005) was found for DBP. There was also a correlation between the 24-hour ABPM and office measurement values (r = 0.57 for SBP and r = 0.24 for DBP; both with p < 0.001).

**Figure 1:**
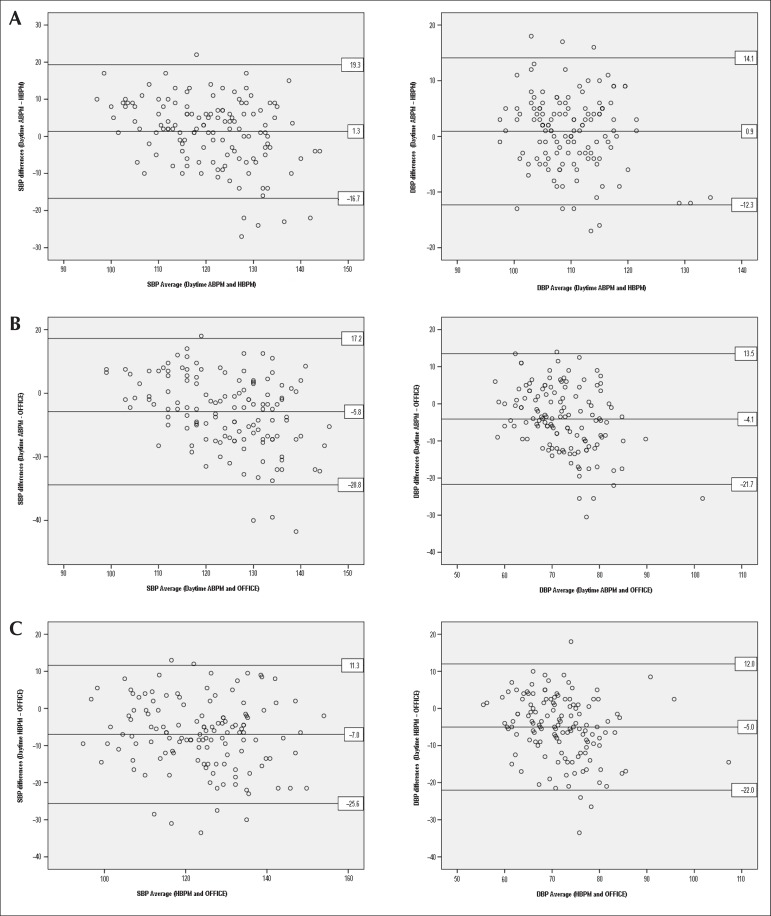
Bland-Altman plot agreement analysis between systolic and diastolic blood pressure (SBP and DBP) values (mmHg) determined by (A) HBPM and daytime ABPM, (B) daytime ABPM and office and (C) HBPM and office.

According to the Bland-Altman graphs, agreement was verified (and no systematic errors were identified) between HBPM and daytime ABPM for SBP and DBP (Figure 1-A); the means of the differences plotted in the central horizontal lines were close to zero (1.3 mmHg for SBP and 0.9 mmHg for DBP). Both daytime ABPM and HBPM agreed with the office measurement values; however, the magnitude was lower: daytime ABPM vs. office, difference in the means of 5.8 mmHg for SBP and 4.1 mmHg for DBP (Figure 1-B); HBPM vs. office, difference in the means of 7.0 mmHg for SBP and 5.0 mmHg for DBP (Figure 1-C).

**Figure 2:**
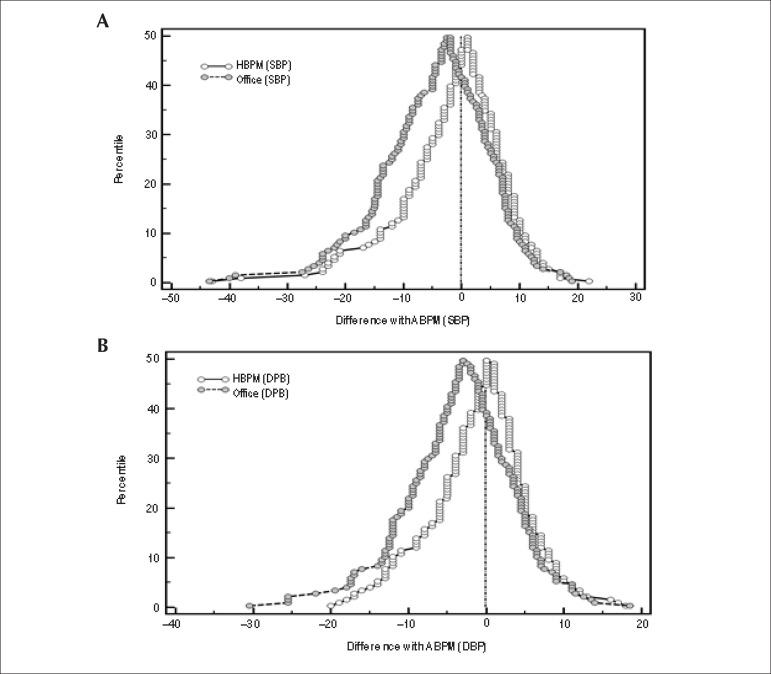
Mountain plots for agreement between (A) systolic blood pressure (SBP) and (B) diastolic blood pressure (DBP) determined by daytime ABPM (reference) and measured by HBPM and office measurement.

From the mountain plots (Figure 2), with daytime ABPM as the reference (axis X), the differences between HBPM and ABPM were generally lower than those observed between the office measurement and daytime ABPM.

## Discussion

This study provides initial information regarding the utilization of HBPM in a Brazilian sample composed exclusively of adolescents, mostly with BP levels higher than normal values. We have identified results similar to those in adults,^9,15,18-21^ for whom office measurements present higher values than HBPM and ABPM for both SBP and DBP. The same phenomenon has already been identified in other studies,^22,-5^ in hypertensive children and adolescents but only with SBP. In contrast to our results, office measurements were similar to HBPM for subjects over 12 years old according to Stergiou et al,^26^ who examined a larger sample (n = 765); however, that study only observed normotensive children and adolescents. There is evidence^26,27^ that the population type (hypertensive vs. normotensives) interferes with the results obtained by office measurement or HBPM.

Another important aspect of BP measurement is the equipment type, and in most studies, the oscillometric method was used. Moreover, analyzing the HBPM protocol is relevant because, currently, there is no consensus on the minimum number of measurements required for pediatric populations. In the present study, we used a total of 24 measurements (with a minimum of 12 measurements) over 6 days, whereas Stergiou et al^26^ opted for a 12-measurement protocol (with a minimum of 2 measurements) over 3 days. This lower number of measurements in HBPM may have contributed to its agreement with the office measurements.

Some studies^23,28-30^ have shown lower HBPM values than daytime ABPM in children and adolescents, which may be explained by the high physical activity levels during childhood, which can increase BP values.

In this study, the result was different, as the BP values measured by HBPM were similar to those obtained by daytime ABPM, which is a commonly observed pattern for adults.^18,19^ This finding is probably related to the fact that the sample consists only of adolescents, who have lower levels of physical activity during the day when compared to children.

Regarding the agreement among methods, a significant number of the studies used the correlation coefficient as an agreement indicator; however, the intrinsic variability of BP renders this index, by itself, inappropriate and requires a variability analysis among measures, such as that accomplished by Bland-Altman plots.^12^ The strength of a correlation between two variables does not necessarily indicate agreement between them. In this study, we showed that the correlation among the three methods was moderate; however, using Bland-Altman plots,^16^ we verified that there was no systematic error among the three methods, particularly between HBPM and daytime ABPM, which showed a difference of zero between the means of the systolic and diastolic pressures. This finding suggests that HBPM may be used as a substitute for ABPM when necessary. Nevertheless, because ABPM is the gold standard, it is still considered the first choice for confirming a diagnosis after detection of high BP by office measurements.

In adults, HBPM shows better reliability and agreement with ABPM than office measurement.^19,31^ In adolescents, we observed a similar phenomenon, which has also been verified in other children and teenage populations, in which HBPM presents better reproducibility than office measurements.^25,32^

The differences between office measurements and the other methods may result in the overestimation of BP values and, consequently, label adolescents as hypertensive when they are actually normotensive. When there is no diagnostic confirmation with other types of evaluation such as HBPM or ABPM, adolescents may be misdiagnosed, with all its social and economic consequences, and even engage in unnecessary treatment by taking medicine. For example, in a study by Hornsby et al,^33^ 44% of the children evaluated as hypertensive by office measurements were reclassified and considered as white coat hypertensive after ABPM.

It has been suggested that office measurements must be a screening method for adolescents and for those who present SBP or DBP values in the > 90th percentile an out-of-office blood pressure method must be performed to confirm the diagnosis. ABPM is the preferred option and HBPM an alternative.^1,27^

HBPM is more comfortable, easy to perform and has a lower cost than ABPM. In this study, daytime ABPM was similar to HBPM. Therefore, HBPM represents an acceptable alternative for a more accurate diagnosis. Nevertheless, when available and financially viable, ABPM should be the first option because it provides a more comprehensive evaluation.

This study was limited by the use of normal values of office measurements proposed for the American population,^1^ as Brazilian studies proposing normal values for adolescents are lacking in the literature. A similar limitation for the HBPM use exists, since the normalcy data for adolescents is based in one study conducted with European students.^26^

Another potential limitation was the inclusion of adolescents enrolled in schools, which excluded adolescents who were out of school. Since the sample studied was obtained from both public and private schools, and since the education system coverage in Brazil is reported as almost universal, this limitation was attenuated.^34^

Longitudinal studies with adolescents that compare the three methods − office, home and ambulatory − and establish adequate normality criteria for different regions of the world are still required.

## Conclusion

HBPM is an alternative option to confirm diagnosis of hypertension with results comparable to ABPM in adolescents from a Brazilian state capital with altered BP values.

## Figures and Tables

**Table 1:** Sample characteristics (n = 133)

	Mean	Standard Deviation	Minimum	Maximum
Age (years)	15.0	±1.6	12	17
Body weight (kg)	65.5	±16.3	37.9	131.5
Height (cm)	167.0	±7.8	149.0	185.5
BMI (kg/m^2^)	23.2	±4.8	15.9	42.5
WC (cm)	75.5	±10.9	58.0	120.0

BMI: body mass index; WC: waist circumference.

## References

[1] National High Blood Pressure Education Program Working Group on High
Blood Pressure in Children and Adolescents (2004). The fourth report on the diagnosis, evaluation, and treatment of
high blood pressure in children and adolescents. Pediatrics.

[2] Muntner P, He J, Cutler JA, Wildman RP, Whelton PK (2004). Trends in blood pressure among children and
adolescents. JAMA.

[3] Lurbe E (2003). Childhood blood pressure: a window to adult
hypertension. J Hypertens.

[4] Magalhaes MG, Oliveira LM, Christofaro DG, Ritti-Dias RM (2013). Prevalence of high blood pressure in Brazilian adolescents and
quality of the employed methodological procedures: systematic
review. Rev Bras Epidemiol.

[5] Bloch KV, Klein CH, Szklo M, Kuschnir MC, Abreu Gde A, Barufaldi LA (2016). ERICA: prevalences of hypertension and obesity in Brazilian
adolescents. Rev Saude Publica.

[6] Lurbe E, Sorof JM, Daniels SR (2004). Clinical and research aspects of ambulatory blood pressure
monitoring in children. J Pediatrics.

[7] Stergiou GS, Alamara CV, Vazeou A, Stefanidis CJ (2004). Office and out-of-office blood pressure measurement in children
and adolescents. Blood Press Monit.

[8] Garrett BN, Salcedo JR, Thompson AM (1985). The role of ambulatory blood pressure monitoring in the
evaluation of adolescent hypertension. Clin Exp Hypertens A.

[9] Mancia G, Fagard R, Narkiewicz K, Redon J, Zanchetti A, Böhm M (2013). 2013 ESH/ESC Guidelines for the management of arterial
hypertension: the Task Force for the Management of Arterial Hypertension of
the European Society of Hypertension (ESH) and of the European Society of
Cardiology (ESC). Eur Heart J.

[10] Lurbe E, Agabiti-Rosei E, Cruickshank JK, Dominiczak A, Erdine S, Hirth A (2016). 2016 European Society of Hypertension guidelines for the
management of high blood pressure in children and
adolescents. J Hypertens.

[11] Pall D, Kiss I, Katona E (2012). Importance of ambulatory blood pressure monitoring in adolescent
hypertension. Kidney Blood Press Res.

[12] Tanner JM (1981). Growth and maturation during adolescence. Nutr Rev.

[13] WHO Expert Committee on Physical Status (1995). The use and interpretation of anthropometry.

[14] Stergiou GS, Yiannes NG, Rarra VC (2006). Validation of the Omron 705 IT oscillometric device for home
blood pressure measurement in children and adolescents: the Arsakion School
Study. Blood Press Monit.

[15] Pickering TG, Shimbo D, Haas D (2006). Ambulatory blood-pressure monitoring. N Engl J Med.

[16] Bland JM, Altman DG (1986). Statistical methods for assessing agreement between two methods
of clinical measurement. Lancet.

[17] Krouwer JS, Monti KL (1995). A simple, graphical method to evaluate laboratory
assays. Eur J Clin Chem Clin Biochem.

[18] Parati G, Stergiou GS, Asmar R, Bilo G, de Leeuw P, Imai Y, ESH Working Group on Blood Pressure Monitoring (2008). European Society of Hypertension guidelines for blood pressure
monitoring at home: a summary report of the Second International Consensus
Conference on Home Blood Pressure Monitoring. J Hypertens.

[19] O'Brien E, Asmar R, Beilin L, Imai Y, Mallion JM, Mancia G, European Society of Hypertension Working Group on Blood Pressure
Monitoring (2003). European Society of Hypertension recommendations for
conventional, ambulatory and home blood pressure measurement. J Hypertens.

[20] Mengden T, Hernandez Medina RM, Beltran B, Alvarez E, Kraft K, Vetter H (1998). Reliability of reporting self-measured blood pressure values by
hypertensive patients. Am J Hypertens.

[21] Mancia G, Bertinieri G, Grassi G, Parati G, Pomidossi G, Ferrari A (1983). Effects of blood-pressure measurement by the doctor on patient's
blood pressure and heart rate. Lancet.

[22] Eicke M, Leumann EP (1989). Ambulatory blood pressure recording in children and adolescents
with a semi-automatic recording device. Helv Paediatr Acta.

[23] Stergiou GS, Alamara CV, Salgami EV, Vaindirlis IN, Dacou-Voutetakis C, Mountokalakis TD (2005). Reproducibility of home and ambulatory blood pressure in children
and adolescents. Blood Press Monit.

[24] Stergiou GS, Nasothimiou E, Giovas P, Kapoyiannis A, Vazeou A (2008). Diagnosis of hypertension in children and adolescents based on
home versus ambulatory blood pressure monitoring. J Hypertens.

[25] Wuhl E, Hadtstein C, Mehls O, Schaefer F, Escape Trial Group (2004). Home, clinic, and ambulatory blood pressure monitoring in
children with chronic renal failure. Pediatr Res.

[26] Stergiou GS, Yiannes NG, Rarra VC, Panagiotakos DB (2007). Home blood pressure normalcy in children and adolescents: the
Arsakeion School study. J Hypertens.

[27] Stergiou GS, Karpettas N, Kapoyiannis A, Stefanidis CJ, Vazeou A (2009). Home blood pressure monitoring in children and adolescents: a
systematic review. J Hypertens.

[28] Soergel M, Kirschstein M, Busch C, Danne T, Gellermann J, Holl R (1997). Oscillometric twenty-four-hour ambulatory blood pressure values
in healthy children and adolescents: a multicenter trial including 1141
subjects. J Pediatr.

[29] Stergiou GS, Alamara CV, Kalkana CB, Vaindirlis IN, Stefanidis CJ, Dacou-Voutetakis C (2004). Out-of-office blood pressure in children and adolescents:
DiPASrate findings by using home or ambulatory monitoring. Am J Hypertens.

[30] Salgado CM, Jardim PC, Viana JK, Jardim Tde S, Velasquez PP (2011). Home blood pressure in children and adolescents: a comparison
with office and ambulatory blood pressure measurements. Acta Paediatr.

[31] Chobanian AV, Bakris GL, Black HR, Cushman WC, Green LA, Izzo JL Jr, Joint National Committee on Prevention, Detection, Evaluation, and
Treatment of High Blood Pressure, National Heart, Lung, and Blood Institute, National High Blood Pressure Education Program Coordinating
Committee (2003). Seventh report of the Joint National Committee on Prevention,
Detection, Evaluation, and Treatment of High Blood Pressure. Hypertension.

[32] Stergiou GS, Salgami EV, Tzamouranis DG, Roussias LG (2005). Masked hypertension assessed by ambulatory blood pressure versus
home blood pressure monitoring: is it the same phenomenon?. Am J Hypertens.

[33] Hornsby JL, Mongan PF, Taylor AT, Treiber FA (1991). 'White coat' hypertension in children. J Fam Pract.

[34] Nascente FM, Jardim TV, Peixoto MD, Carneiro CS, Mendonça KL, Póvoa TI (2016). Sedentary lifestyle and its associated factors among adolescents
from public and private schools of a Brazilian state capital. BMC Public Health.

